# The NLRP3 inflammasome in urogenital cancers: structure, dual function, and therapeutic potential

**DOI:** 10.3389/fonc.2025.1593774

**Published:** 2025-07-11

**Authors:** Weiming Sun, Ruiting Ma, Rui Zhao, Jiaju Tian, Wenhui Liu

**Affiliations:** ^1^ The First Hospital of Lanzhou University, Endocrinology Department, Lanzhou, China; ^2^ The First Clinical Medical College, Lanzhou University, Lanzhou, China

**Keywords:** NLRP3, inflammasome, urogenital cancer, activation mechanism, targeted therapy

## Abstract

The NLRP3 inflammasome is a multi-subunit complex consisting of the NLRP3 receptor protein, apoptosis-associated speck-like protein (ASC), and the effector protein caspase-1. The activation mechanisms of the NLRP3 inflammasome are diverse and complex, primarily involving both classical and non-classical pathways. The activation process is generally considered to occur in two stages: the first, “priming,” which involves the initial immune signaling trigger, followed by the second stage, “activation,” during which further signaling leads to the formation and functional activation of the NLRP3 inflammasome. Upon activation, the NLRP3 inflammasome activates caspase-1, triggering the maturation and release of downstream pro-inflammatory cytokines, such as IL-18 and IL-1β, which in turn promote the inflammatory response. In recent years, an increasing body of research has highlighted the close association between the NLRP3 inflammasome and its downstream signaling pathways with the development and progression of urogenital system cancers. These studies suggest that the NLRP3 inflammasome may serve as a potential therapeutic target, holding significant clinical application value in cancer treatment. Despite the growing number of related studies, a comprehensive and systematic review summarizing the role of the NLRP3 inflammasome in urogenital cancers and its potential therapeutic strategies is still lacking. This review aims to provide an extensive overview of the composition and activation mechanisms of the NLRP3 inflammasome, explore its involvement in urogenital system cancers, delve into related targeted therapy strategies, and discuss future research directions, with the goal of offering valuable insights for researchers in this field.

## 1 Introduction

In recent years, urogenital system tumors, including prostate cancer, bladder cancer, cervical cancer, endometrial cancer, ovarian cancer, and kidney cancer, have garnered significant attention due to their major threat to public health. Among these, prostate cancer ranked as the second most common cancer globally in 2022 and is reported to be one of the leading causes of cancer-related mortality among men in Western countries (1). For women, cervical cancer ranks as the fourth most common cancer in both incidence and mortality. Ovarian cancer (OC), recognized as the most fatal gynecological malignancy, remains a leading cause of cancer-related death among women (2).

Inflammation occurs as a self-protective and reparative response when tissues are damaged by toxins, bacteria, infections, or other causes; thus, it represents a fundamental defense mechanism against external stimuli. While normal inflammatory responses are a beneficial aspect of the immune system, recent studies increasingly suggest that inflammation is often associated with cancer initiation and progression (3). In fact, approximately 25% of carcinogenic factors are attributed to infection and inflammation, which play roles at various stages of cancer, including initiation, promotion, malignant transformation, invasion, and metastasis (4). Cellular and molecular mediators of inflammation regulate tumor growth by modulating adaptive immune responses, adjusting the balance of immune cell types and signaling within the tumor microenvironment, thereby exerting either tumor-supportive or tumor-suppressive effects. For instance, the “injury and regeneration” model proposes that damage induced by pathogens or pro-inflammatory cytotoxins can lead to the proliferation of prostate cells, resulting in epithelial lesions known as “proliferative inflammatory atrophy” (PIA), a precursor to prostate cancer (5).

The innate immune system serves as a critical first line of defense against inflammation. It identifies infections and disruptions in cellular homeostasis, triggering responses to eliminate pathogens and repair tissue damage (6). Inflammasomes are multiprotein complexes that assemble within cells in response to pathogen-associated molecular patterns (PAMPs) and damage-associated molecular patterns (DAMPs). Early studies established inflammasomes as essential components of innate immunity (7). Among them, the NLRP3 inflammasome has been extensively studied in recent years (8). Comprising the receptor protein NLRP3, the adaptor protein ASC, and the effector protein caspase-1, the NLRP3 inflammasome detects external stimuli, recruiting and activating the caspase-1 precursor. This activation cleaves the precursors of cytokines IL-1β and IL-18, releasing their active forms into the extracellular space to induce inflammatory responses and trigger pyroptosis (9). Although recent studies have examined its role and clinical relevance in cancer development, findings remain limited and inconsistent (10). This review focuses on summarizing the structure and activation mechanisms of the NLRP3 inflammasome and elucidates its role in urogenital system tumors, such as prostate and bladder cancers, highlighting its potential as a therapeutic target.

## 2 NLRP3 inflammasome

### 2.1 Structure of the NLRP3 inflammasome

Inflammasomes are multiprotein complexes assembled by cytoplasmic pattern recognition receptors (PRRs), playing a crucial role in innate immunity. Currently, five primary types of inflammasomes have been identified: NLRP1, NLRP3, NLRC4, IPAF, and AIM2. Among these, the NLRP3 inflammasome is the most extensively studied. Like other inflammasomes, the NLRP3 inflammasome is composed of three components: the receptor protein NLRP3, the adaptor protein ASC (apoptosis-associated speck-like protein), and the effector protein caspase-1.

Pattern recognition receptors (PRRs) play a pivotal role in immunology, with NLRP3 being a key member of this group. It is capable of recognizing specific molecular patterns on pathogens, apoptotic cells, and damaged cells (11, 12). Compared to other inflammasomes, the NLRP3 inflammasome is the most extensively studied (13). Its activation is triggered by a wider range of stimuli, making it more complex than other inflammasomes (14). NLRP3 plays a crucial role in the progression of various inflammatory diseases (15). In the context of cancer, inflammation is involved in initiation, promotion, malignant transformation, invasion, and metastasis. Beyond the general pro-inflammatory effects observed in other inflammasomes (16), NLRP3 also exhibits inhibitory effects on both inflammation and cancer progression, likely due to its unique “pyroptotic pathway” (17). As a result, the NLRP3 inflammasome has emerged as a promising therapeutic target for inflammatory diseases and NLRP3-related small molecule inhibitors are currently undergoing clinical trials (18). Understanding its structure and activation mechanisms is therefore of critical importance.

The NLRP3 protein comprises three main domains: an N-terminal pyrin domain (PYD), a central nucleotide-binding and oligomerization domain (NACHT) with ATPase activity, and a C-terminal leucine-rich repeat (LRR) domain (19). The PYD domain is responsible for interacting homotypically with the PYD domain of ASC, initiating downstream assembly. The NACHT domain possesses ATPase activity, which allows it to bind and hydrolyze ATP, thereby driving the oligomerization of the NLRP3 protein—a crucial prerequisite for inflammasome assembly (20). The LRR domain is involved in ligand recognition and in binding regulatory proteins, such as NEK7.The NLRP3 inflammasome shares a common assembly mechanism with other ASC-dependent inflammasomes (21), involving two sequential nucleation-induced polymerization steps. These domains work in concert to sense intracellular danger signals and mediate the assembly of the inflammasome. Upon stimulation by pathogen-associated molecular patterns (PAMPs) or damage-associated molecular patterns (DAMPs), the NACHT domain drives the oligomerization of NLRP3 and exposes its PYD domain. This exposed PYD domain then recruits the adaptor protein ASC via PYD–PYD interactions. Subsequently, ASC uses its C-terminal CARD domain to recruit pro-caspase-1, which also contains a CARD domain. During this recruitment, pro-caspase-1 is cleaved to remove its N-terminal prodomain, generating the active form of caspase-1. These interactions ultimately lead to the formation of a complete NLRP3 inflammasome complex (22).

**Figure 1:**
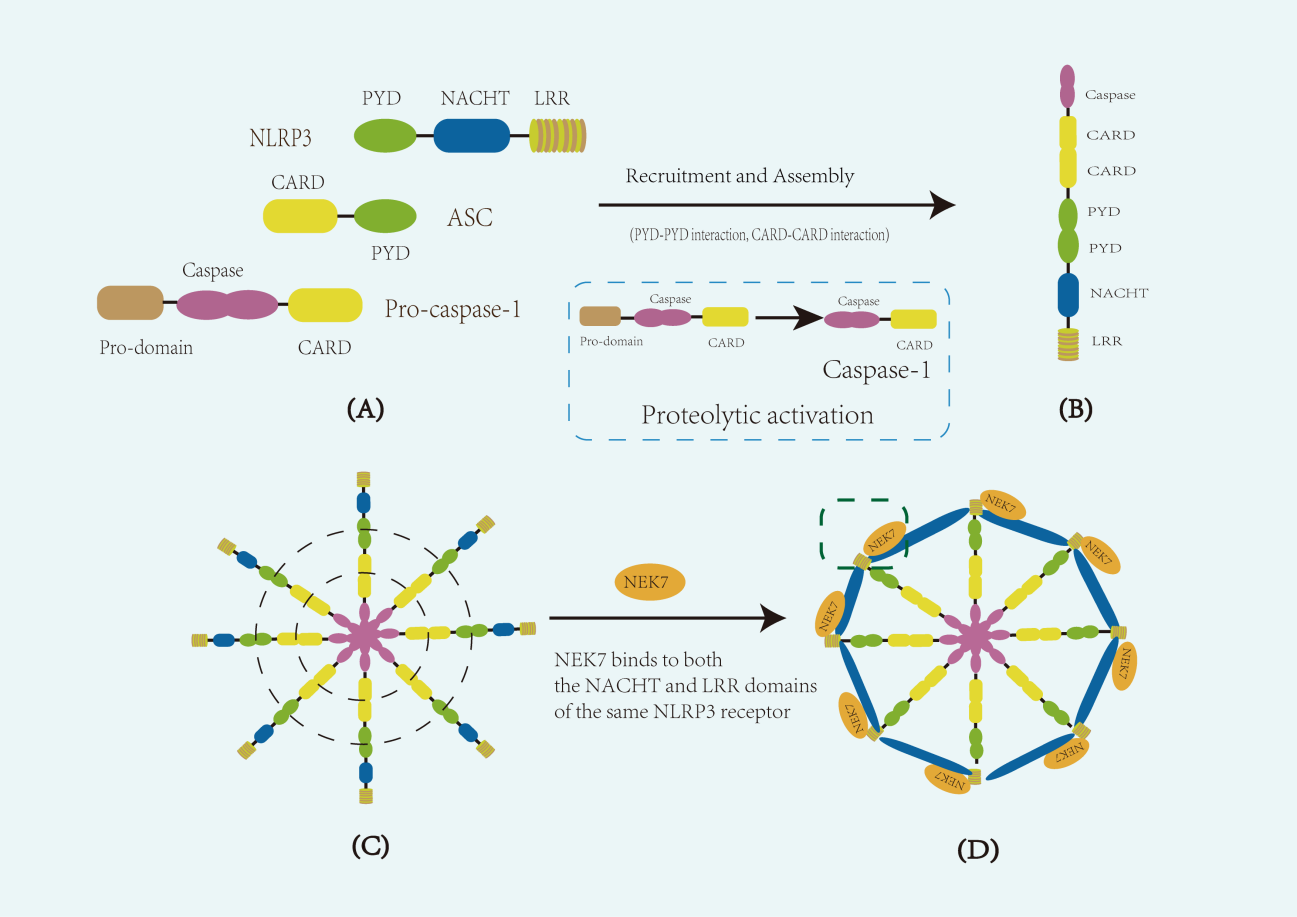
Schematic representation of the composition and structure of the NLRP3 inflammasome. **(A)** Diagram of the core components of the NLRP3 inflammasome. The inflammasome consists of the sensor protein NLRP3, the adaptor protein ASC (apoptosis-associated speck-like protein containing a CARD), and the effector protein pro-caspase-1. NLRP3 comprises three main domains: an N-terminal pyrin domain (PYD), a central NACHT domain with ATPase activity responsible for oligomerization, and a C-terminal leucine-rich repeat (LRR) domain. **(B)** Diagram of a single assembled NLRP3 inflammasome. Upon activation, the NACHT domain mediates NLRP3 oligomerization, exposing the PYD domain, which recruits ASC through homotypic PYD–PYD interactions. ASC then recruits pro-caspase-1 via CARD–CARD interactions. During this process, pro-caspase-1 undergoes autocatalytic cleavage to remove its N-terminal prodomain, generating active caspase-1. **(C)** Diagram of multiple aggregated inflammasome complexes. The aggregation of multiple inflammasome units enhances downstream signaling. Dashed lines in panel **(C)** indicate key domain–domain interactions, including PYD–PYD interactions between NLRP3 and ASC, and CARD–CARD interactions between ASC and pro-caspase-1. **(D)** Diagram of the functional disc-like inflammasome complex. In the activated state, NEK7 binds to both the NACHT and LRR domains of the same NLRP3 molecule, stabilizing inflammasome assembly and promoting the formation of an active disc-like structure. The dashed box in panel **(D)** indicates the binding interface between NEK7 and NLRP3.

With advancements in electron microscopy, Researchers have gained a deeper understanding of the conformational changes and dynamic assembly process of the NLRP3 inflammasome (**Figure 1**). The structure of its NACHT and LRR domains was initially revealed by cryo-electron microscopy (cryo-EM) of the NLRP3-NEK7 complex (23) and has since been corroborated by additional cryo-EM studies. In the inactive state, ADP stably binds the NACHT domain, whose tripartite subdomainsedTE.DATA ote><Cite domain (NBD), helical domain 1 (HD1), and winged-helix domain (WHD)n-hel a tightly packed conformation that maintains the autoinhibited state (24). Upon activation, ATP replaces ADP, inducing conformational rearrangements that expose the PYD domain for oligomerization. The C-terminal LRR domain, which is thought to serve as a scaffold for autoregulation and ligand sensing, folds back onto the NACHT region in the inactive state and likely extends outward during activation. These changes allow NEK7 to bind to the LRR and NACHT regions of the same NLRP3 molecular, stabilizing the active conformation. Ultimately, this structural reorganization facilitates ASC filament formation via PYD–PYD interactions, and the recruitment of pro-caspase-1 via CARD–CARD interactions. Upon recruitment, pro-caspase-1 undergoes autocatalytic cleavage to remove its N-terminal prodomain, generating active caspase-1. Together, these events drive the assembly of the disc- or wheel-shaped inflammasome complex (25).

### 2.2 Activation of the NLRP3 inflammasome

The persistent activation of the NLRP3 inflammasome is associated with the induction of chronic inflammation (26), which is widely recognized as a significant risk factor for cancer development (27). Therefore, a comprehensive understanding of its activation mechanisms is of great significance for elucidating the pathogenesis of cancer and developing therapeutic strategies.

It is generally accepted that the activation of the NLRP3 inflammasome occurs in two functionally complementary yet processually distinct phases: priming and activation. The priming phase primarily regulates the expression of key molecules such as NLRP3, pro-IL-1β, and pro-IL-18, preparing them for subsequent assembly, while the activation phase triggers inflammasome assembly and downstream signaling (28). The constitutive expression level of NLRP3 alone is insufficient to activate the inflammasome (29, 30), and the “two-hit” activation hypothesis is commonly evaluated *in vitro* using lipopolysaccharides (LPS) (31). Physiological NLRP3 oligomers are thought to detect diverse signals to initiate inflammasome activation (32). Recent studies have highlighted the complexity of the priming step, which involves transcriptional and post-translational mechanisms (33). The priming step acts on Toll-like receptors (TLRs), leading to downstream activation of NF-κB (34, 35), which upregulates NLRP3 and other pro-inflammatory factors, such as pro-IL-1β and pro-IL-18. Post-translational modifications, such as ubiquitination and phosphorylation, also play critical roles in NLRP3 inflammasome activation. Ubiquitination exerts a dual regulatory effect on the NLRP3 inflammasome: on one hand, it promotes NLRP3 activation through deubiquitination, while on the other hand, specific forms of ubiquitination can facilitate NLRP3 degradation, thereby maintaining its inactive state (36). E3 ubiquitin ligases are key negative regulators in this process. Previous studies have confirmed that the E3 ligase TRIM31 directly interacts with NLRP3, mediating K48-linked polyubiquitination, thereby directing NLRP3 to the proteasomal degradation pathway (37). Additionally, another E3 ligase complex, SCF-FBXL2, continuously ubiquitinates NLRP3 in the resting state, inhibiting its aberrant activation and promoting its stable degradation (38). Previous studies have revealed that protein kinase D (PKD) phosphorylates NLRP3 at the Golgi apparatus (39), with phosphorylation serving as a critical priming event that influences NLRP3 deubiquitination (40) and subsequently affects NLRP3 inflammasome activation (41). Additionally, Schroder and colleagues first demonstrated that the priming of the NLRP3 inflammasome with short lipopolysaccharide (LPS) can occur independently of NLRP3 protein expression (42).

During the activation step, NLRP3 detects pathogen-associated molecular patterns (PAMPs) and damage-associated molecular patterns (DAMPs), including toxins, ATP (43), microbial products, and particulate matter (44), which promote inflammasome activation and lead to the cleavage of pro-IL-1β and pro-IL-18 by caspase-1, producing mature IL-1β and IL-18 (45). This triggers a cascade of inflammatory responses, and activated caspase-1 cleaves gasdermin-D (GSDMD) into an N-terminal fragment. The fragment subsequently accumulates on the inner side of the cell membrane, forming pores approximately 10–20 nanometers in diameter, disrupting membrane integrity and thereby inducing pyroptosis. Due to the absence of a conventional signal peptide, the mature IL-1β and IL-18 cannot be secreted through the classical endoplasmic reticulum-Golgi pathway but instead rely on membrane pores for passive release into the extracellular space (46). Studies have found that these pores are negatively charged, which selectively facilitates the release of negatively charged cytokines like IL-1β and IL-18, distinguishing them from their inactive precursors (47).

**Figure 2:**
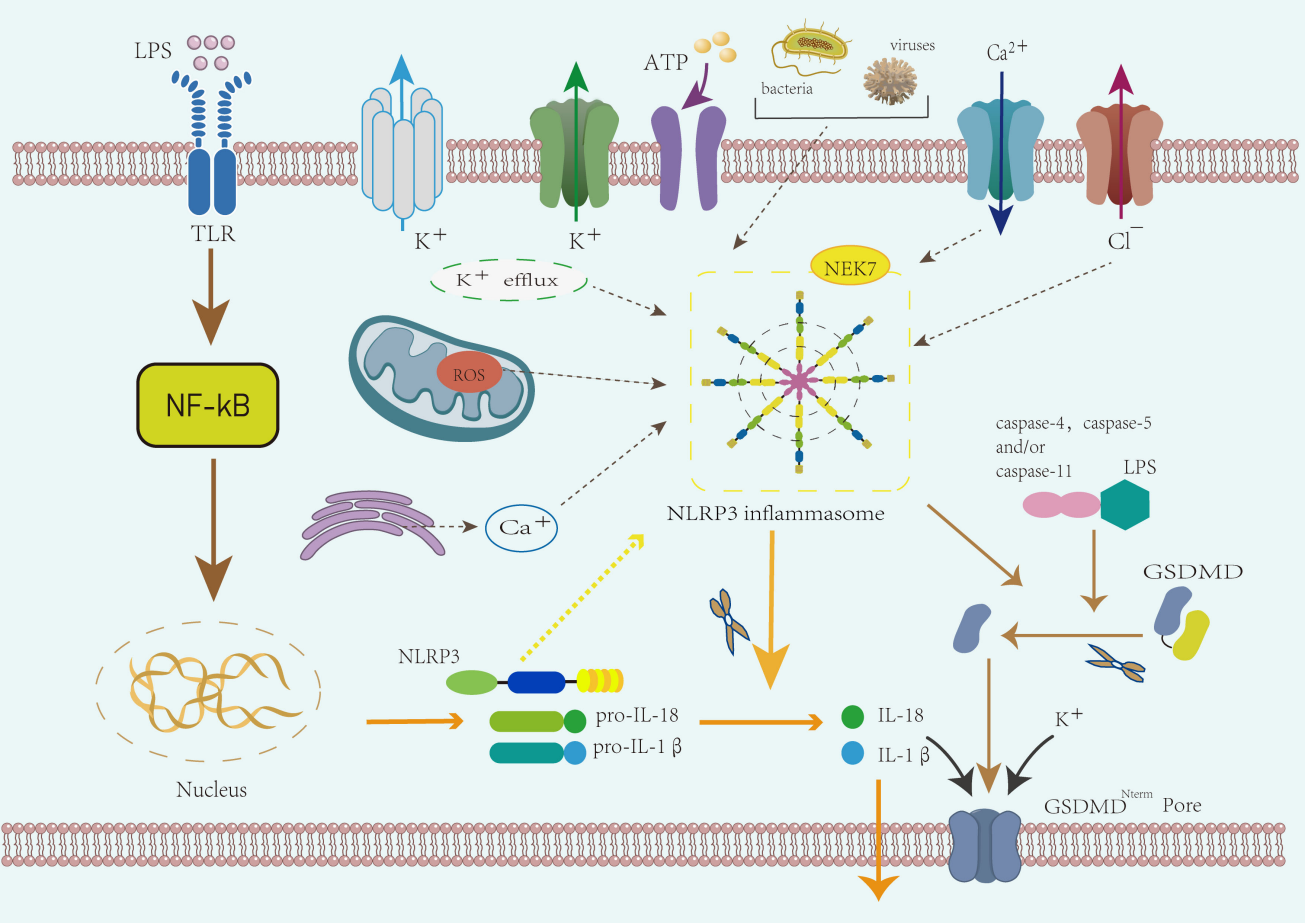
Mechanism of NLRP3 inflammasome activation. The activation of the NLRP3 inflammasome proceeds in two stages: priming and activation. During the priming phase, the activation of Toll-like receptors (TLRs) triggers NF-κB signaling, leading to the upregulation of NLRP3, pro-IL-1β, and pro-IL-18. The activation phase is triggered by external stimuli such as bacteria, ATP, or other damage-associated signals. This phase involves both canonical and non-canonical activation mechanisms. In the canonical pathway, potassium efflux, mitochondrial dysfunction, and ROS generation contribute to inflammasome assembly, with NEK7 acting as a key regulator. Activated caspase-1 processes pro-IL-1β and pro-IL-18 into mature cytokines and induces pyroptosis via GSDMD. The non-canonical activation pathway involves caspase-11 (in mice) or caspase-4/5 (in humans), which directly bind LPS and cleave GSDMD, contributing to cell death and inflammation.

The activation mechanisms of the NLRP3 inflammasome are currently classified into canonical and non-canonical pathways (48, 49) (**Figure 2**). The canonical activation primarily involves changes in ion gradients, such as K^+^efflux (50), Cl^−^efflux (51), mitochondrial dysfunction, and reactive oxygen species (ROS) generation (52). Studies have identified Nek7 as a key downstream regulator of inflammasome activation following K^+^efflux (14, 53). K^+^ efflux, mediated by the two-pore domain weakly rectifying K channel 2 (TWIK2) (54), is essential for the subsequent post-translational processing and maturation of pro-IL-1β (55). Therefore, K^+^ efflux serves as a common pathway for the majority of activation mechanisms. The non-canonical activation mechanism is dependent on murine caspase-11 or human caspase-4 and -5 (56), which directly bind lipopolysaccharide (LPS) via their CARD domains (57). After binding, caspase-11 dimerizes and acquires self-cleaving activity (58). LPS also activates caspase-4 and -5, leading to gasdermin D cleavage, which promotes pyroptosis and caspase-1–dependent activation of NLRP3 (59). Interestingly, research suggests that murine caspase-11 and human caspase-4 and -5 may not directly mature pro-IL-1β, but instead induce K^+^ efflux, thereby triggering the canonical NLRP3 activation pathway (60). The non-canonical activation of the NLRP3 inflammasome may play a critical role in certain cancer types (61). Activation of caspase-11 and caspase-4 not only directly contributes to NLRP3 activation but may also influence the dynamics of the tumor immune microenvironment (62), thereby promoting immune evasion in tumors (63).

The activation of the NLRP3 inflammasome is regulated by various proteins. For instance, double-stranded RNA-dependent protein kinase (PKR) interacts with inflammasome components to modulate its activation (64). The orphan nuclear receptor Nur77, a lipopolysaccharide (LPS)-binding protein found in macrophage lysates (65), binds directly to LPS via its C-terminus, facilitating its association with NLRP3. Notably, the interaction between Nur77 and NLRP3 requires the simultaneous presence of both LPS and double-stranded DNA (dsDNA), elucidating how the NLRP3 inflammasome responds to LPS and is activated by caspase-11. HOIL-1L is essential for NLRP3/ASC inflammasome assembly and linear ubiquitination of ASC, identifying it as a novel substrate of the LUBAC complex (66). As an alternative pathway, human apolipoprotein C3 (ApoC3) activates the NLRP3 inflammasome in human monocytes through caspase-8 and the dimerization of Toll-like receptors 2 and 4 (67). Additionally, studies indicate that endoplasmic reticulum stress also promotes NLRP3 inflammasome activation (68).

The activation of the NLRP3 inflammasome, along with the associated NF-κB pathway and cytokines such as IL-1β and IL-18, has been shown to play a significant role in various inflammatory diseases. Reported inflammatory diseases influenced by this pathway primarily include cardiovascular diseases (such as atherosclerosis, abdominal aortic aneurysm, myocardial infarction, dilated cardiomyopathy, diabetic cardiomyopathy, and heart failure) (69), neurological disorders (such as Alzheimer’s disease and Parkinson’s disease) (70), autoimmune diseases, diabetes, and several types of cancer.

Two mechanisms have been proposed to explain how the activated NLRP3 inflammasome is terminated. The first mechanism relies on caspase-1, a key component of the NLRP3 inflammasome, which undergoes a second autolysis to generate p20/p10 subunits. This process leads to the loss of its enzymatic activity, thereby preventing the cleavage of downstream substrates, pro-IL-1β and pro-IL-18, effectively terminating the inflammasome-mediated inflammatory cascade (71). The second mechanism of termination involves autophagy. Studies have shown that activated NLRP3 inflammasomes typically migrate along microtubules, where they are recognized in the cytoplasm and sequestered by a double-membrane structure to form autophagosomes. These autophagosomes subsequently fuse with lysosomes, leading to degradation of the inflammasomes (72). Together, these two termination mechanisms ensure that the activity of the NLRP3 inflammasome remains within an appropriate range, thereby preventing excessive inflammatory responses that could damage host tissues. They play a critical role in maintaining immune homeostasis and in preventing the onset and progression of chronic inflammation-related diseases.

The activation and termination of the NLRP3 inflammasome are finely regulated by various signaling molecules and regulatory mechanisms. Both classical and non-classical pathways synergistically drive the inflammatory response, while negative feedback mechanisms, such as caspase-1 auto-cleavage and autophagy, prevent excessive activation (73). This complex network not only maintains immune homeostasis but also plays a crucial role in the development of various cancers (74), laying the foundation for further exploration of its tumor-related functions and therapeutic potential.

## 3 The role of the NLRP3 inflammasome in various urogenital tumors

Due to the close commonalities between different tumors of the urogenital system, they are often studied as a unified group. From the perspective of embryonic origin, organs such as the kidneys, bladder, and ovaries all originate from the mesoderm, and therefore, their cells share certain similarities during early development. Anatomically, urogenital system tumors are typically located in the lower abdominal cavity, and during their growth, they often influence each other or undergo local metastasis. For example, studies have shown that prostate cancer patients who receive radiotherapy may, in rare cases, be diagnosed with muscle-invasive bladder cancer (MIBC) (75). The kidneys and gonads are both involved in hormone synthesis and secretion, and during tumorigenesis, they often exhibit similar dysregulation in hormone secretion. Additionally, urine contains free proteins, peptides, exosomes, DNA, and other components, making it possible to detect urogenital system tumors through urinary biomarkers (76).

With the in-depth research on TRP channels, the role of their downstream NLRP3 inflammasome and related pathways in tumor cell proliferation and metastasis has become relatively clear (77). In recent years, studies have also indicated that the NLRP3 inflammasome plays a dual role in the progression of urogenital tumors (**Table 1**), particularly in prostate and bladder cancers (78–80). On one hand, NLRP3-related genes are upregulated in cancer cells, leading to the activation of the NLRP3 inflammasome, which, through caspase-1 cleavage, generates pro-inflammatory cytokines, namely, mature IL-1β and IL-18 (81). These cytokines have significant roles in the development of urogenital tumors. Mature IL-1β recruits immune cells to sites of infection; however, IL-1β overexpression is associated with various autoimmune diseases and may contribute to carcinogenesis. Meanwhile, mature IL-18 enhances the activity of NK and T cells, potentially influencing tumor progression. Obesity is an established risk factor for cancers in several urogenital sites, including the endometrium, kidney, and ovaries (82). The NLRP3 inflammasome may contribute to the progression of these cancers by amplifying this risk. Specifically, the NLRP3 inflammasome can activate caspase-1, leading to the production of IL-1β and IL-18, which promote the metastasis and progression of urogenital tumors. Additionally, the NLRP3 inflammasome may enhance the impact of other carcinogenic risk factors associated with urogenital cancers (83).

**Table 1:** The function of NLRP3 across cancer types.

Type of cancer	Source of experimental evidence	Outcome	Suggested mechanism	References
Pro-tumorigenic Role				
Prostate cancer	GWAS summary data across 8 populations	IL-18 identified as a prostate cancer risk factor	Maturation of IL-18	(91)
	RWPE-1 prostate epithelial cells stimulated by *T. vaginalis*	T. vaginalis induces IL-1β production	ROS and K^+^ efflux activate NLRP3 inflammasome	(94)
	PC3-EVs act on THP-1 macrophages	IL-1β secretion correlates with tumor staging	NLRP3 inflammasome activation cascade in tumor cells	(92)
Bladder cancer	Genetic polymorphism studies (175 BC patients)	IL-18 polymorphism increases BC risk	Genetic susceptibility via inflammasome variants	(93)
	NLRP3 polymorphisms in smokers/drinkers	NLRP3 polymorphisms linked to increased tumor size and metastasis	Environment–gene interaction	(79)
Renal cell carcinoma	MassARRAY and urinary arsenic analysis	NLRP3 polymorphism increases RCC risk	Arsenic exposure upregulates NLRP3 expression	(108)
	KIRC tumor tissue analysis	NLRP3 mRNA upregulated; promoter hypomethylation	NLRP3 expression correlates with overall survival and immune cell infiltration	(109)
Ovarian cancer	OvCa cells	NLRP3 overexpression promotes EMT and tumor progression	NLRP3 modulates EMT markers (E/N-cadherin)	(117)
	TCGA analysis and survival data	Elevated NLRP3 expression correlates with reduced survival	NLRP3 correlates with inflammation-linked transformation in EAOC	(118, 119)
	CCK-8 assay (proliferation), western blot (EMT & PI3K/AKT markers), luciferase assay, Kaplan–Meier survival analysis	NLRP3 overexpression associated with poor prognosis via miR-22 suppression	NLRP3 counteracts miR-22’s inhibitory effects on proliferation and EMT	(120)
	Clinical study on advanced epithelial ovarian cancer	IL-1 associated with impaired T-cell responses	NLRP3-induced IL-1 impairs antitumor immunity	(121)
Cervical cancer	Genetic association study of NLRP3 SNP (rs10754558)	Rs10754558 associated with increased cervical cancer risk	Modulation of HPV-related immune responses	(126)
	LPS-stimulated normal cervical epithelial cells	LPS promotes proliferation, invasion, and cytokine release	NLRP3 and caspase-4 mediated cytokine production	(129)
	Mechanistic analysis of NLRP3/IL-1β/Smad2/3 signaling	EMT promotes tumor cell migration and metastasis	NLRP3/IL-1β pathway activates Smad2/3 signaling to drive EMT	(130)
Endometrial cancer	Immunohistochemistry and immunoblotting in EMC tissues and cell lines	NLRP3, caspase-1, GSDMD overexpressed in tumor tissues	Upregulation of pyroptosis-related proteins in cancerous tissue	(131, 132)
	Case–control study on obesity, T2D, NAFLD in 98 participants	Inflammasome-related cytokines upregulated in obesity-related EMC	NLRP3–IL-1β axis mediates obesity-induced inflammation contributing to EMC progression	(135)
Anti-tumorigenic Role				
Prostate cancer	Immune profiling of tumor microenvironment	NLRP3 activation linked to immune suppression of tumor progression	IL-18 promotes Th2 response and M2 macrophage polarization	(97)
Bladder cancer & Renal cell carcinoma	Chemotherapy-induced pyroptosis study	NLRP3 activation promotes CD8^+^ T cell–mediated antitumor response	ATP release activates NLRP3 and IL-1β–IL-1R signaling in dendritic cells	(113)
Renal cell carcinoma	BRD4 inhibition in RCC cells	Induces pyroptosis, suppresses EMT	NF-κB–NLRP3–caspase-1–GSDMD axis	(114)
Ovarian cancer	Electron microscopy + mechanistic assays	CA induces pyroptosis and inhibits tumor growth	CASP4/TXNIP–NLRP3–GSDMD pathway mediates CA-induced pyroptosis	(124)
	Functional study on PFKFB3 in OvCa cells	PFKFB3 enhances metastasis by inhibiting pyroptosis	NLRP3 suppression reduces pyroptosis and promotes Warburg effect	(125)
Endometrial cancer	Bioinformatics and macrophage coculture assays	NLRP3 depletion promotes tumor progression and impairs immunity	NLRP3 regulates macrophage polarization and ROS-mediated antitumor defense	(137–139)
	IHC of pyroptosis markers in 351 EMC patients	Pyroptosis markers predict clinical outcomes	High GSDMD and low CHMP4B associated with favorable recurrence-free survival	(140)

**Figure 3:**
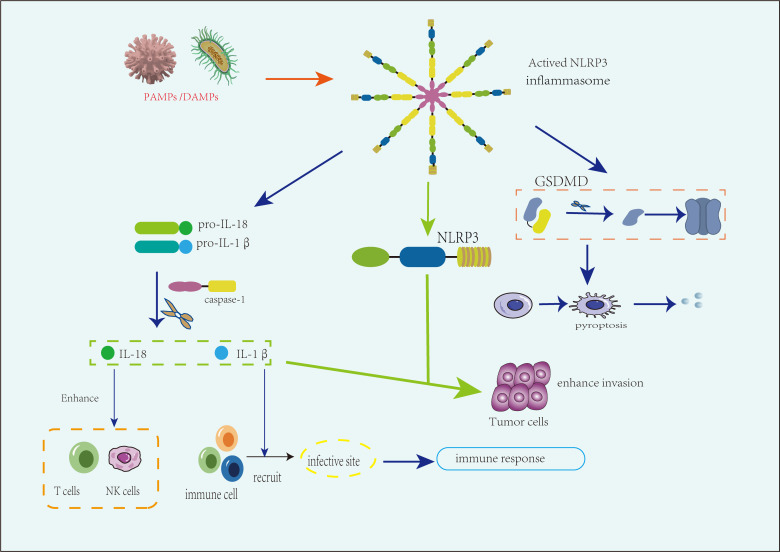
The role of the NLRP3 inflammasome in various urogenital tumors. The NLRP3 inflammasome plays a dual role in the progression of urogenital tumors. On the one hand, activation by pathogen-associated molecular patterns (PAMPs) and damage-associated molecular patterns (DAMPs) triggers inflammasome formation, leading to the caspase-1-mediated production of IL-1β and IL-18. These cytokines promote tumor metastasis and progression by enhancing inflammation and immune evasion, particularly under immunosuppressive conditions such as in advanced tumor stages or obesity-related risks. On the other hand, in immune-activated environments, IL-18 enhances the activity of natural killer (NK) cells and T cells, while IL-1β recruits immune cells to the tumor site. Additionally, the inflammasome induces tumor suppression through pyroptosis, particularly via gasdermin D (GSDMD)-mediated cell death, which can limit tumor growth and spread.

On the other hand, the NLRP3 inflammasome may inhibit tumor progression by modulating immune responses and inducing pyroptosis in cancer cells. The NLRP3 inflammasome has the capacity to activate inflammatory responses, and one study has highlighted its contribution to infection-related immune mediation (84). Additionally, the GSDMD pathway, triggered by NLRP3 activation, can induce pyroptosis in cancer cells, thereby suppressing cancer growth and invasion (**Figure 3**). However, current research on the inhibitory role of the NLRP3 inflammasome in urogenital tumors remains limited, with some studies presenting conflicting conclusions (85).

The tumor microenvironment (TME) is crucial in tumor progression, interacting continuously with tumor cells and playing a decisive role in tumor initiation, development, metastasis, and response to therapy. The TME comprises a variety of immune cells, fibroblasts, macrophages, neutrophils, endothelial cells, pericytes, and other stromal components, forming a complex and dynamically interacting network with tumor cells (86). It has been established that the NLRP3 inflammasome can modulate the TME by regulating inflammatory status and immune cell phenotypes, thereby influencing tumor behavior (87). Notably, the dual role of NLRP3 appears to be closely related to the composition and activation state of immune cells within the tumor microenvironment (TME). In immunosuppressive conditions, excessive expression of IL-1β downstream of NLRP3 activation may promote tumor progression by exacerbating chronic inflammation and facilitating immune evasion. In contrast, under immune-activated conditions, NLRP3-mediated pyroptosis—particularly through gasdermin D (GSDMD)-dependent pore formation—can induce cancer cell death and enhance antitumor immune responses (88). This context-dependent functional divergence highlights the importance of considering the specific characteristics of the TME when evaluating the role of NLRP3 across different tumor types.

### 3.1 The role of the NLRP3 inflammasome in prostate cancer

Prostate cancer (PCa) is a highly heterogeneous and complex cancer with a high prevalence and significant variability in clinical progression among individuals. Current treatment options often carry substantial risks of complications.

Research on the role of the NLRP3 inflammasome in prostate cancer progression has increased in recent years, primarily focusing on its promoting effects, although findings remain contradictory, indicating the need for further investigation. NLRP3-related genes are upregulated in prostate cancer, with NLRP3 inflammasome protein overexpressed (89); this overexpression correlates with clinical parameters such as cancer staging and lymph node infiltration. Contradictorily, a study analyzing NLRP3 expression in human prostate tissue found that NLRP3 immunostaining was heterogeneous, with no significant difference observed between adjacent benign and malignant tissues. This discrepancy may be attributed to several factors, including tumor heterogeneity, the use of adjacent benign tissues that might already be affected by an inflammatory tumor microenvironment, and the reliance on immunohistochemistry alone, which reflects protein expression but not inflammasome activation status. Moreover, the study lacked functional validation, subgroup analysis by tumor grade or stage, and assessment of cytokine output, which limits its ability to capture the dynamic pro- or anti-tumor role of NLRP3 in prostate cancer (90). A Mendelian randomization study, using GWAS summary data on 35 interleukins from large-scale proteomic studies across eight independent populations of European descent and data on 23 common cancers from the FinnGen Consortium, identified IL-18 as a risk factor for prostate cancer (91–93). Another study used non-cancerous prostate cell line PNT2 treated with extracellular vesicles (EVs) isolated from advanced prostate cancer PC3 cells (PC3-EVs) as a model to assess caspase-1–mediated IL-1β maturation after 24 hours of EV treatment. In a separate study, live *Trichomonas vaginalis* (T. vaginalis) stimulation of the prostate epithelial cell line RWPE-1 led to increased mRNA and protein expression of IL-1β, NLRP3, ASC, and caspase-1. Silencing of NLRP3 and caspase-1 reduced T. vaginalis–induced IL-1β secretion, and the specific NF-κB inhibitor Bay 11–7082 suppressed IL-1β production while inhibiting the expression of caspase-1, ASC, and NLRP3 proteins. These findings suggest that T. vaginalis induces NLRP3 inflammasome formation in human prostate epithelial cells through ROS and potassium efflux, leading to IL-1β production (94). In a prostate cancer model, mice lacking IL-1β exhibited impaired tumor progression and angiogenesis (95). Furthermore, analysis of cytokine expression and the migration and proliferation profiles of PC3 cells indicated that PC3-EVs impact differentiated THP-1 macrophages, showing that PC3 cells contain an active NLRP3 inflammasome cascade and secrete IL-1β, which is associated with tumor staging (92). Since IL-18 and IL-1β are produced in large quantities during NLRP3 inflammasome activation, current findings indicate that the NLRP3 inflammasome and its downstream pathways may promote prostate cancer progression.

The NLRP3 inflammasome may exert protective effects on prostate cancer by modulating the tumor microenvironment and immune response. The prostate tumor microenvironment primarily consists of T cells, B cells, neutrophils, macrophages, myeloid-derived suppressor cells, and fibroblasts (96, 97). IL-18 mediates neutrophil maturation and local infiltration, and it induces T cells to differentiate into a Th2 phenotype. Th2 cells secrete IL-4 and IL-13, which promote macrophage polarization to the M2 phenotype, mediating anti-inflammatory effects (98). Thus, NLRP3 activation enables mature IL-18 to function as an anti-inflammatory factor. Moreover, K^+^ efflux, one of the activation pathways of the NLRP3 inflammasome, has been identified as an early event in the apoptosis of prostate cancer cells (99), suggesting that the NLRP3 inflammasome may induce tumor cell apoptosis through this mechanism.

### 3.2 The role of the NLRP3 inflammasome in bladder cancer and renal cell carcinoma

Bladder cancer (BC) and renal cell carcinoma (RCC) are common urological tumors. In China, bladder cancer has the highest incidence among urological cancers, while in Western countries, it ranks second only to prostate cancer. Both the incidence and aggressiveness of BC are significantly higher in men than in women (100), Known risk factors for BC include smoking, gender, age, and genetic predisposition. The incidence of RCC varies globally, with smoking, obesity, and hypertension being the most strongly associated risk factors. The mortality rate for RCC is approximately 30-40%, and it is more common in men than in women (101).

The NLRP3 inflammasome, along with IL-18 and IL-1β, plays a significant role in promoting the development of bladder cancer (BC) and renal cell carcinoma (RCC). Studies have demonstrated that NLRP3 mRNA and protein levels are markedly elevated in BC cells compared to normal urothelial cells (102). NLRP3 polymorphisms are associated with increased BC risk, tumor size, and lymph node metastasis, particularly among smokers and drinkers (79). A genotyping study involving 175 high-grade BC patients and 207 healthy controls showed that IL-18 gene polymorphisms increase BC risk (93), a finding corroborated by additional research (103). In T24 cells, Wnt signaling—a key pathway in BC development—is activated by high levels of IL-1β, while Wnt signaling, in turn, upregulates IL-1β expression. This paracrine Wnt/IL-1β signaling feedback loop enhances the invasive phenotype of BC cells (104, 105).Arsenic is a known carcinogen implicated in the development of RCC and BC (106, 107). A study using the Agena Bioscience MassARRAY platform identified 15 NLRP3 gene polymorphism sites, and arsenic species concentrations in urine were measured via HPLC-HG-AAS. This study found a significant dose-dependent association between arsenic exposure and RCC risk, with NLRP3 polymorphisms increasing susceptibility to RCC in individuals with elevated total urinary arsenic (108). Additionally, arsenic exposure was shown to upregulate NLRP3 expression. In a study on kidney clear cell carcinoma (KIRC), NLRP3 expression was significantly elevated in tumor tissues, and NLRP3 mRNA expression appeared to be regulated by promoter methylation. A significant negative correlation was observed between NLRP3 promoter methylation and NLRP3 expression, which was further associated with the clinicopathological features, overall survival, and immune cell infiltration in KIRC (109).

Substantial evidence indicates a positive correlation between the incidence of type 2 diabetes (T2DM), obesity, and bladder cancer (110). In one study, obesity (HR = 1.76, 95% CI: 1.36-2.28) was associated with a significantly higher recurrence rate of bladder cancer compared to normal-weight patients, with stratified analysis revealing that the recurrence risk was higher in women than in men (HR = 1.17, 95% CI: 1.05-1.31). Obesity is also a major risk factor for RCC. Studies have demonstrated that NLRP3 knockout in mice can prevent obesity-induced inflammasome activation in adipose tissue and the liver, enhancing insulin signaling. In obese mice, NLRP3 deletion reduced IL-18 and interferon-γ (IFN-γ) expression in adipose tissue, increased naive T cell counts, and decreased effector T cell counts (111). Additionally, under oxidative stress induced by the tumor microenvironment, thioredoxin-interacting protein (TXNIP) interacts with the NLRP3 inflammasome (112), leading to IL-1β maturation and secretion, which correlates with postoperative RCC recurrence. This further suggests that the NLRP3 inflammasome and its associated cytokines contribute to RCC progression.

However, the NLRP3 inflammasome is essential for antitumor adaptive immunity in bladder cancer (BC) and renal cell carcinoma (RCC), and its pyroptosis pathway has demonstrated potent antitumor effects. Dying tumor cells treated with chemotherapy release ATP, which activates the NLRP3 inflammasome and IL-1β–IL-1 receptor (IL-1R) signaling in dendritic cells, driving an effective CD8 T-cell response against transplantable tumor cells (113). Another study measured BRD4 expression in RCC cells and normal human renal tubular epithelial cell lines, finding significantly elevated BRD4 expression in RCC cells. Inhibition of BRD4 suppressed cell proliferation and epithelial-mesenchymal transition (EMT) through activation of the NF-κB–NLRP3–caspase-1 pyroptosis pathway, demonstrating its antitumor effects (114). Additionally, lower NLRP3 levels were observed in RCC tumor samples, suggesting that NLRP3 may act as a tumor suppressor in RCC (115). Therefore, the NLRP3 inflammasome plays a notable dual role in the progression of BC and RCC.

### 3.3 The role of the NLRP3 inflammasome in ovarian cancer

Ovarian cancer (OvCa) is a common gynecological malignancy with subtle early symptoms and limited treatment options after diagnosis (116). It is characterized by a relatively low incidence but a high mortality rate.

Overexpression of NLRP3 and other related proteins is a marker associated with the progression of ovarian cancer. For example, one study reported NLRP3 overexpression in ovarian cancer cells, and bioinformatics analysis further explored the role of NLRP3 in promoting OvCa progression (117). Additionally, NLRP3 silencing inactivated the inflammasome and inhibited epithelial-mesenchymal transition (EMT) by upregulating E-cadherin and downregulating vimentin, N-cadherin, and fibronectin. A study investigating endometriosis-associated ovarian cancer (EAOC) analyzed 18 genes related to the inflammasome complex to examine their correlation with patient survival. The findings revealed a statistically significant association between high NLRP3 expression levels and poor prognosis, suggesting that dysregulated inflammasome activity may play a key role in the malignant transformation of endometriosis. Furthermore, NLRP3 signaling and persistent sterile inflammation may mark the early stages of ovarian cancer development (118). Data from The Cancer Genome Atlas program comparing NLRP3 expression in pan-cancer and normal tissues showed higher expression in ovarian cancer, correlating with reduced overall survival (119). Another study measured cell proliferation using the CCK-8 assay, evaluated protein expression of EMT and PI3K/AKT pathway biomarkers through western blotting, assessed luciferase activity via luciferase assays, and used the Kaplan-Meier method to evaluate overall survival in ovarian cancer. The results indicated low miR-22 expression and NLRP3 overexpression in ovarian cancer tissues and cells, with miR-22 downregulation associated with poor prognosis. NLRP3 partially countered miR-22’s regulatory effects on cell proliferation and EMT in ovarian cancer cells (120). Additionally, Maccio et al. demonstrated that IL-1 is associated with impaired T-cell responses in women with advanced epithelial ovarian cancer (121), suggesting that interleukins activated by NLRP3 may promote ovarian cancer progression by influencing immune responses. However, a comparative study assessing NLRP3 (NOD-like receptor protein 3), caspase-1, IL-1β, and IL-18 mRNA and protein expression in hen and human OvCa found increased protein expression of caspase-1, IL-1β, and IL-18 in surface epithelium, tumor cells, and immune cells. No significant difference in NLRP3, caspase-8, or caspase-11 mRNA levels was observed between tumor and non-tumor ovarian tissues, indicating that the specific NLR sensor involved remains to be identified (122).

Obesity increases the risk of ovarian cancer. In a study on obesity, adipose tissue from obese participants (n = 186) showed significantly higher NLRP3 gene expression compared to non-obese participants (n = 84) (SMD 1.07; 95% CI, 0.27–1.87) (123). Additionally, a pooled analysis of adipose tissue IL-1β data from four studies indicated that IL-1β gene expression levels were significantly elevated in 88 obese participants compared to 39 non-obese controls (SMD 0.56; 95% CI, 0.13–0.99). These findings suggest an association among the NLRP3 inflammasome, obesity, and ovarian cancer.

Meanwhile, pyroptosis induced by the NLRP3 inflammasome can inhibit tumor progression. A study using morphological analysis via transmission electron microscopy showed that ovarian cancer cells treated with citric acid (CA) exhibited typical pyroptosis characteristics. Further mechanistic analysis indicated that CA induces pyroptosis in ovarian cancer through the CASP4/TXNIP-NLRP3-Gasdermin-D (GSDMD) pathway, thereby inhibiting ovarian cancer cell growth and presenting a potential therapeutic approach (124). Another study on PFKFB3 in ovarian cancer found that PFKFB3 promotes ovarian cancer metastasis by suppressing the NLRP3 axis, reducing pyroptosis, and enhancing the Warburg effect. This indirectly confirms the protective role of NLRP3-mediated pyroptosis in preventing ovarian cancer metastasis and progression (125).

### 3.4 The role of the NLRP3 inflammasome in uterine malignancies

Uterine malignancies pose a significant threat to women’s health, with poor prognoses when diagnosed at advanced stages. Treatment options for targeted therapy in advanced or recurrent cases remain limited, and incidence rates have been rising in recent years. These malignancies include cervical cancer (CC) and endometrial cancer (EMC).

The relationship between the NLRP3 inflammasome and uterine malignancies is complex. Pontillo et al. reported that a variant of the NLRP3 gene, rs10754558, is associated with HPV resistance and shows a statistically significant correlation with cervical cancer risk (126). Caspase-1, a component of the NLRP3 inflammasome, has previously been shown to enhance lipid metabolism, and its activation is necessary for intracellular Chlamydia growth in cervical epithelial cells (127). In one study, inflammasome activation was first analyzed in cocultured cervical cancer cell lines and healthy donor monocytes, with *in vitro* findings then compared to data from a public CC patient database. Results indicated that IL-1β expression was elevated in CC compared to normal cervical tissue, and patients with high IL-1β expression had shorter overall survival, suggesting that the CC microenvironment may activate inflammasomes and IL-1β release in surrounding monocytes, potentially worsening prognosis (128). Another study stimulated normal cervical epithelial cells with LPS, leading to enhanced proliferation and invasion, alongside increased expression of inflammatory cytokines (IL-1β, IL-6, and TNF-α) as well as NLRP3 and caspase-4, further underscoring the role of NLRP3 and IL-1β in cancer progression (129). In CC, the NLRP3/IL-1β/Smad2/3 signaling pathway promotes epithelial-mesenchymal transition (EMT), a key process in metastasis where epithelial cells acquire mesenchymal characteristics, enhancing motility and migratory capacity (130). Additionally, immunohistochemistry (IHC) and protein immunoblotting studies have shown overexpression of NLRP3, caspase-1, and GSDMD in human endometrial cancer tissues and cell lines, with moderate to strong positive staining for these proteins in tumor sections (131, 132). Estrogen has also been shown to enhance NLRP3, pro-IL-1β, and IL-1β expression, thereby promoting endometrial cancer progression through increased NLRP3 activation (133).

Obesity increases the risk of endometrial cancer (134), and the NLRP3-IL-1β pathway plays a crucial role in adipose tissue (AT)-induced inflammation and the development of obesity-related comorbidities. In a case-control study involving 98 participants, it was demonstrated that obesity (P < 0.01), obesity-related type 2 diabetes (T2D) (P < 0.01), and non-alcoholic fatty liver disease (NAFLD) (P < 0.05) increased the expression of various inflammasome components as well as the expression and release of IL-1β and IL-18 in AT (135). In summary, the NLRP3 inflammasome promotes endometrial cancer progression.

The NLRP3-related pathway can induce pyroptosis, modulate immune responses, and consequently inhibit the progression of uterine malignancies. miRNA-214 promotes pyroptosis in cervical cancer cells by enhancing NLRP3 expression, thus inhibiting cancer progression (136). NLRP3 also plays a significant role in regulating macrophage polarization, oxidative stress, and immune response against endometrial cancer (EMC). A bioinformatics analysis indicated that NLRP3 levels in intratumoral macrophages in EMC are significantly lower than in normal endometrial macrophages. Depletion of NLRP3 in M2-polarized macrophages promoted the growth, invasion, and metastasis of cocultured EMC cells, while NLRP3 depletion in M1-polarized macrophages reduced phagocytic potential, thereby weakening immune defense against EMC (137). Additionally, The NLRP3 inflammasome can enhance the production of ROS, particularly in macrophages (138). ROS are highly reactive molecules that can induce oxidative stress, resulting in substantial damage to cellular components. Moreover, ROS are involved in the regulation of the tumor cell cycle, thereby affecting tumor cell survival and migration (137). Therefore, ROS production regulated by NLRP3 shows promise as a potential therapeutic target for EMC, indicating that NLRP3 loss may impact the development of new EMC treatment strategies (139). Another study assessed the expression of four pyroptosis-related molecules (NLRP3, cleaved caspase-1 p20, cleaved gasdermin D, and CHMP4B) in 351 endometrial cancer patients via immunohistochemistry and analyzed their associations with clinical, pathological, and survival outcomes. It concluded that cleaved caspase-1 p20 is an independent predictor of poor prognosis in EMC, while high cleaved gasdermin D and low CHMP4B expression were associated with favorable recurrence-free survival (RFS) (140).

## 4 Targeted therapeutic strategies related to the NLRP3 inflammasome

Given the significant role of the NLRP3 inflammasome and related pathways in the development and progression of urogenital tumors, research on targeted therapeutic strategies involving the NLRP3 inflammasome has increased considerably in recent years (141).

Inhibiting NLRP3 and its downstream pathways is a primary focus in targeted therapeutic strategies (**Table 2**). Small-molecule inhibitors have become a key approach in these strategies (142). One of the most extensively studied inhibitors is MCC950, a specific small-molecule inhibitor of the NLRP3 pathway (143). MCC950 directly targets the NACHT domain of NLRP3, blocking ASC oligomerization, ATP hydrolysis (144), and stabilizing the inactive conformation of NLRP3 (145). Oridonin (Ori), the primary active compound in the traditional Chinese herb Rabdosia rubescens, has anti-inflammatory properties. Ori forms a covalent bond with cysteine 279 in the NACHT domain of NLRP3, thereby disrupting the NLRP3-NEK7 interaction and inhibiting inflammasome assembly and activation—a mechanism that has been confirmed in ovarian cancer (146). Similarly, CY-09 binds directly to the ATP-binding motif within the NACHT domain, inhibiting NLRP3 ATPase activity and thereby preventing inflammasome assembly and activation (147). The anticancer agent entrectinib (ENB) has also shown significant efficacy in mouse models of NLRP3 inflammasome-related diseases. Studies have demonstrated that ENB binds directly to arginine 121 (R121) of NEK7, blocking the NEK7-NLRP3 interaction and inhibiting inflammasome assembly and activation (148). Ibrutinib, a Bruton’s tyrosine kinase (BTK) inhibitor, blocks NLRP3 inflammasome activation in macrophages, inhibits IL-1β secretion, and reduces inflammation and organ fibrosis (149). Betaine, also known as trimethylglycine, is a naturally occurring compound in animals, plants, and microorganisms. It has shown anti-inflammatory effects and potential anticancer benefits in humans by modulating sulfur amino acid metabolism, counteracting oxidative stress, inhibiting NF-κB activity, and suppressing NLRP3 inflammasome activation. Betaine also regulates energy metabolism and reduces endoplasmic reticulum stress and apoptosis (150). The P2X7 receptor, critical for inflammasome activation, induces the maturation and release of pro-inflammatory cytokines (e.g., IL-1β and IL-18) and promotes the production of reactive nitrogen and oxygen species, caspase activation, and apoptosis. Sennoside A (SenA), a novel caspase-1 inhibitor, inactivates caspase-1 in a P2X7-dependent manner to suppress NLRP3-mediated inflammation (151). Estrogen-related receptor alpha (ERRα), a central regulator of cellular energy metabolism linked to poor cancer prognosis, enhances glycolytic metabolism and targets the NLRP3/caspase-1/GSDMD pathway to regulate pyroptosis in endometrial cancer (152). Resveratrol (RSV) inhibits tumor cell proliferation, migration, and invasion *in vivo* and *in vitro*, promoting apoptosis in renal cell carcinoma. Further mechanistic analysis reveals that RSV suppresses tumor progression in renal cell carcinoma by downregulating NLRP3 (153). Additionally, downregulation of NLRP3 enhances gemcitabine sensitivity in ovarian cancer (GRC) cells, potentially overcoming drug resistance and improving therapeutic efficacy (154).

**Table 2:** NLRP3 inhibitors.

NLRP3 inhibitors	Targets	Preclinical/clinical status	Cancer type studied	Reference
MCC950	NACHT domain of NLRP3	Preclinical (*in vitro* & *in vivo*)	Not yet investigated in oncology (inflammatory disease)	(144, 145)
Oridonin	NACHT domain of NLRP3; disrupts NLRP3–NEK7	Preclinical (*in vitro*, cancer model)	Ovarian cancer	(146)
CY-09	ATP-binding motif within the NACHT domain	Preclinical (*in vitro*)	Not yet investigated in oncology (inflammatory disease)	(147)
Entrectinib	NEK7-NLRP3 interaction	Preclinical (mouse model, *in vivo*)	Not yet investigated in oncology (NLRP3-related mouse disease model)	(148)
Ibrutinib	Bruton’s tyrosine kinase (mediatie the phosphorylation of NLRP3)	Preclinical (macrophage inflammation model)	Not yet investigated in oncology (macrophage inflammation/fibrosis)	(149)
Betaine	NLRP3 and mature caspase-1	Preclinical (metabolic inflammation studies)	Not yet investigated in oncology (inflammation/oxidative stress)	(150)
Sennoside A	Caspase-1 (P2X7-dependent inhibition)	Preclinical (inflammatory model)	Not yet investigated in oncology (P2X7/caspase-1 pathway)	(151)
Resveratrol	Downregulates NLRP3	Preclinical (*in vitro* & *in vivo*)	Renal cell carcinoma	(153)
Ulinastatin	RhoA/ROCK/NLRP3 pathway	Preclinical (prostate cancer cell line)	Prostate cancer	(156)
Suramin	Suppresses NLRP3 expression	Preclinical (Pkd1 miRNA mouse model)	Not yet investigated in oncology (NLRP3-related renal pathology model)	(157)
Thalidomide	caspase-1	Clinical (combination with docetaxel)	Prostate cancer	(159)
Parthenolide	caspase-1	Preclinical (anti-inflammatory studies)	Not yet investigated in oncology (inflammatory disease)	(161)
Anakinra	IL-1β	Preclinical (glioblastoma invasion model)	Glioblastoma	(162)
Mitomycin C	immunogenic cell death	Approved (NMIBC treatment)	Non-muscle-invasive bladder cancer	(163)
Carvedilol	NLRP3-caspase-1-ASC inflammasome	Preclinical (prostate cancer cell lines)	Prostate cancer	(165)

Adiponectin-induced autophagy suppresses LPS-induced inflammasome activation in macrophages (155). Ulinastatin improves the malignant progression of prostate cancer cells by blocking the RhoA/ROCK/NLRP3 pathway (156). Suramin, an antiparasitic drug with potent anti-purinergic properties, has shown effectiveness in a transgenic Pkd1 microRNA mouse model, where suramin treatment significantly reduced kidney cyst density, cell proliferation, and macrophage infiltration. Mechanistic studies confirmed that suramin suppresses NLRP3 expression, thereby inhibiting tumor progression (157). Thalidomide, an effective anti-inflammatory drug, inhibits caspase-1 activity (158). Experimental studies comparing docetaxel combined with thalidomide versus docetaxel alone demonstrated thalidomide’s antitumor activity in prostate cancer treatment (159). Parthenolide, widely used as an herbal treatment for various inflammatory diseases (160), has been shown to directly inhibit the protease activity of caspase-1 (161). The IL-1 receptor antagonist anakinra can suppress IL-1β-mediated inflammatory responses, thereby reducing the invasiveness of glioblastoma (162). Mitomycin C (MMC), an effective treatment for non-muscle-invasive bladder cancer (NMIBC), promotes immunogenic cell death (ICD) and tumor protection *in vivo*. MMC-induced ICD relies on metabolic reprogramming in tumor cells to enhance oxidative phosphorylation (163). Studies have shown that cocrystals downregulate inflammatory markers such as NLRP3 and caspase-1, and the antifungal drug FCN also exhibits anticancer activity (164). Carvedilol (CVL), a β-adrenergic receptor antagonist, promotes NF-κB nuclear translocation through the NLRP3-caspase-1-ASC inflammasome, inducing pyroptosis in prostate cancer (PCa) cells and establishing a foundation for the application of β-adrenergic antagonists in PCa treatment (165).

NLRP3 ubiquitination presents a potential therapeutic target for disease treatment. The deubiquitinating enzyme BRCC3 is a key regulator of NLRP3 activity (166). The vitamin D receptor (VDR) can physically bind to NLRP3, blocking the interaction between NLRP3 and BRCC3. When VDR inhibits BRCC3-mediated NLRP3 deubiquitination, subsequent NLRP3 activation is also suppressed (167). Therefore, the vitamin D receptor (VDR) serves as a negative regulator of NLRP3 oligomerization and activation.

The epigenetic regulation of inflammasome-related genes may serve as a potential target for further studies on molecular mechanisms that modulate inflammatory pathways. Research has shown that the expression of various NLRP genes (e.g., NLRP3, NLRP4, NLRP9) and miRNAs targeting these genes varies in urinary sediment from bladder cancer patients compared to healthy controls (168, 169). miRNAs are critical regulators of NLRP3 inflammasome activity (170), and assessing the expression levels of inflammasome-related genes and their regulatory miRNAs may provide a promising diagnostic tool for bladder cancer.

## 5 Conclusion and future directions

Although substantial progress has been made in understanding the NLRP3 inflammasome, its multifaceted and context-dependent role in urogenital system tumors remains incompletely understood. It has been implicated in both pro-tumor and anti-tumor processes, depending on tumor type and microenvironmental context. These functional heterogeneities highlight the urgent need for further research to elucidate its precise mechanisms and therapeutic relevance in cancer.

Current research has several limitations, mainly reflected in the contradictions in conclusions, limitations of external effects, and challenges in clinical application. First, many research findings are contradictory, likely due to differences in cancer staging, clinical parameters, and other influencing factors. Therefore, future studies should incorporate a broader range of variables, considering cancer type-specific staging and clinical parameters. Second, existing studies predominantly focus on specific populations (e.g., individuals of European descent), which limits the generalizability of the findings to other ethnic groups, genders, or age groups. Future research should prioritize multi-center, cross-ethnic studies to ensure the applicability of results across diverse populations. Finally, while there is a wealth of data from basic and animal studies, there is a lack of large-scale clinical trial data, particularly involving diverse ethnic groups, which limits the clinical translation of NLRP3-targeted therapeutic strategies.

The significant role of the NLRP3 inflammasome in cancer provides new directions for therapeutic strategies. Future studies could design large-scale cohort studies to assess the diagnostic value of combining NLRP3 inflammasome levels with classic tumor biomarkers such as AFP and CA-125. These studies should include diverse patient populations and utilize quantitative PCR or ELISA assays to measure NLRP3 inflammasome activation, aiming to validate their diagnostic accuracy through receiver operating characteristic (ROC) curve analysis for early cancer detection. Given the potential of combining NLRP3-targeted therapies with immune checkpoint inhibitors, clinical trials should be designed to evaluate the safety and efficacy of this combination therapy in patients with advanced urogenital cancers. Randomized controlled trials (RCTs) could focus on overall survival (OS) and progression-free survival (PFS) as primary endpoints, with biomarkers of immune response (e.g., cytokine profiles, immune cell infiltration) as secondary endpoints. The trials should stratify patients based on NLRP3 expression levels to assess whether higher NLRP3 activity correlates with a better response to immunotherapy.

Moreover, for urogenital system cancers, particularly ovarian and prostate cancers, DNA damage response inhibitors, such as PARP inhibitors, have shown promise as therapeutic options. To further explore this, future studies should design preclinical and clinical trials investigating the role of the NLRP3 inflammasome in modulating DNA repair mechanisms in cancer cells. Specifically, *in vitro* studies could focus on measuring NLRP3 expression and activation in response to DNA damage induced by agents like cisplatin or radiation in ovarian and prostate cancer cell lines. In parallel, clinical trials could assess the combination of PARP inhibitors with NLRP3-targeted therapies in patients with high NLRP3 expression, evaluating treatment efficacy by monitoring tumor progression, DNA repair markers (e.g., γ-H2AX), and patient response rates. Furthermore, the role of tRNA level changes and their crosstalk with the tumor microenvironment (TME) in tumor development has garnered increasing attention in recent years. Future studies should examine how tRNA modifications influence NLRP3 inflammasome activation by performing RNA sequencing in tumor tissues with varying levels of NLRP3 expression. Using high-throughput screening, researchers could identify specific tRNA modifications that correlate with NLRP3 activation. The dynamic interactions with the TME could be assessed using advanced imaging techniques such as multiplex immunohistochemistry to visualize NLRP3 localization and tRNA alterations *in situ*, providing a better understanding of how these interactions contribute to tumor progression.

Additionally, the NLRP3 inflammasome may be closely associated with cancer cell metabolic pathways, particularly the key metabolic processes such as oxidative stress, glycolysis, and fatty acid metabolism. These pathways not only support the rapid proliferation and growth of tumor cells but may also offer new insights into the dual role of NLRP3 inflammasome in cancer. To explore the interactions between NLRP3 and cancer cell metabolism, future research should combine transcriptomic analysis of NLRP3 expression in various cancer cell lines with metabolic profiling using mass spectrometry or nuclear magnetic resonance (NMR). *In vivo* studies could involve xenograft models treated with NLRP3 inhibitors and metabolic modulators, with analysis of tumor growth, metabolic shifts, and treatment response. Multi-omics data integration could help identify novel metabolic pathways regulated by NLRP3 that contribute to tumor progression.

In conclusion, the NLRP3 inflammasome plays a pivotal role in the development and progression of urogenital system tumors, with its regulatory mechanisms involving tumor cell proliferation, apoptosis, immune evasion, and inflammatory signaling. Its activation generally follows a well-recognized two-step model—priming and activation—leading to the release of pro-inflammatory cytokines such as IL-1β and IL-18. These factors can promote tumor growth and invasion or trigger pyroptotic cell death, depending on the cellular context. This review has summarized current advances in understanding the composition, activation mechanisms, functional heterogeneity, and therapeutic targeting potential of the NLRP3 inflammasome in urogenital cancers. Future studies should further elucidate its upstream signaling pathways, downstream effectors, interactions with classical tumor biomarkers, and roles in cancer cell metabolism. Such efforts will enhance diagnostic precision and accelerate the clinical translation of immunotherapies and targeted therapies centered on NLRP3, ultimately offering more precise and effective treatment strategies for cancer patients.
